# Exploring gender differences in depressive symptoms and life conditions among first-generation Vietnamese migrants in Germany

**DOI:** 10.1016/j.jmh.2026.100447

**Published:** 2026-10-15

**Authors:** Laura Elisabeth Tuturea, Eric Hahn, Luisa Eilinghoff, Kerem Böge, Marco Zierhut, Thi Minh Tam Ta

**Affiliations:** a Department of Psychiatry and Neurosciences, Charité – Universitätsmedizin Berlin, Campus Mitte, Charitéplatz 1, Berlin, 10117, Germany; b Department of Psychiatry and Neurosciences, Charité – Universitätsmedizin Berlin, Campus Benjamin Franklin, Hindenburgdamm 30, Berlin, 12203, Germany; c DZPG , German Center for Mental Health, Campus Mitte, Charitéplatz 1, Berlin, 10117, Germany; d Department of Psychology, Medical University Brandenburg, Fehrbelliner Str. 38, Neuruppin, 16816, Germany

**Keywords:** Gender, Depression, Migrants, Mental health, Vietnamese, Female, Germany

## Abstract

**Background** Gender plays a crucial role in shaping migrants' mental health. Yet gender differences remain insufficiently studied**.**

**Objective** The study seeks to provide a more nuanced understanding of gender differences in depressive symptoms and life conditions among first-generation Vietnamese migrants in Germany, including potential indirect pathways linking gender to depressive symptoms.

**Methods** A nonclinical sample of 326 first-generation Vietnamese migrants in Germany completed a survey assessing sociodemographic (e.g., age, financial status), migration-related (e.g., language proficiency, cultural orientation), and psychological variables, including depressive symptoms and psychosocial stressors (e.g., health concerns, relationship conflicts, traumatic memories). Analyses included gender comparisons, correlations with depressive symptoms, and an exploratory mediation model using structural equation modelling.

**Results** Clinically significant depressive symptoms were approximately three times as prevalent among women as among men (15.4% vs. 4.8%). Women also reported higher levels of psychosocial stress, particularly health concerns, appearance concerns, family caregiving stress, financial concerns, and perceived lack of support, whereas men demonstrated higher employment rates, better German language proficiency, greater educational attainment in Germany, and a stronger orientation toward German culture. The mediation model identified a significant indirect effect via psychosocial stress.

**Discussion** Findings underscore the importance of addressing gender-specific challenges among migrants. Identified intertwined factors such as language proficiency and education in Germany, employment, financial status, orientation toward German culture, and especially psychosocial stressors, including social support, health concerns, financial concerns, and family caregiving stress, are suggested to be a focus of further research, policies and targeted mental health services.

## Highlights

• Women report more stressors: health, appearance, caregiving, finances, support.
• Men report higher host-culture orientation, language, education, employment.
• Depression (PHQ-9 ≥ 10): 15.4% female vs. 4.8% male Vietnamese migrants
• Psychosocial stressors mediated gender-depression gap.
• Nonclinical community sample enhances applicability beyond patients.

## Graphical abstract

**Figure:**
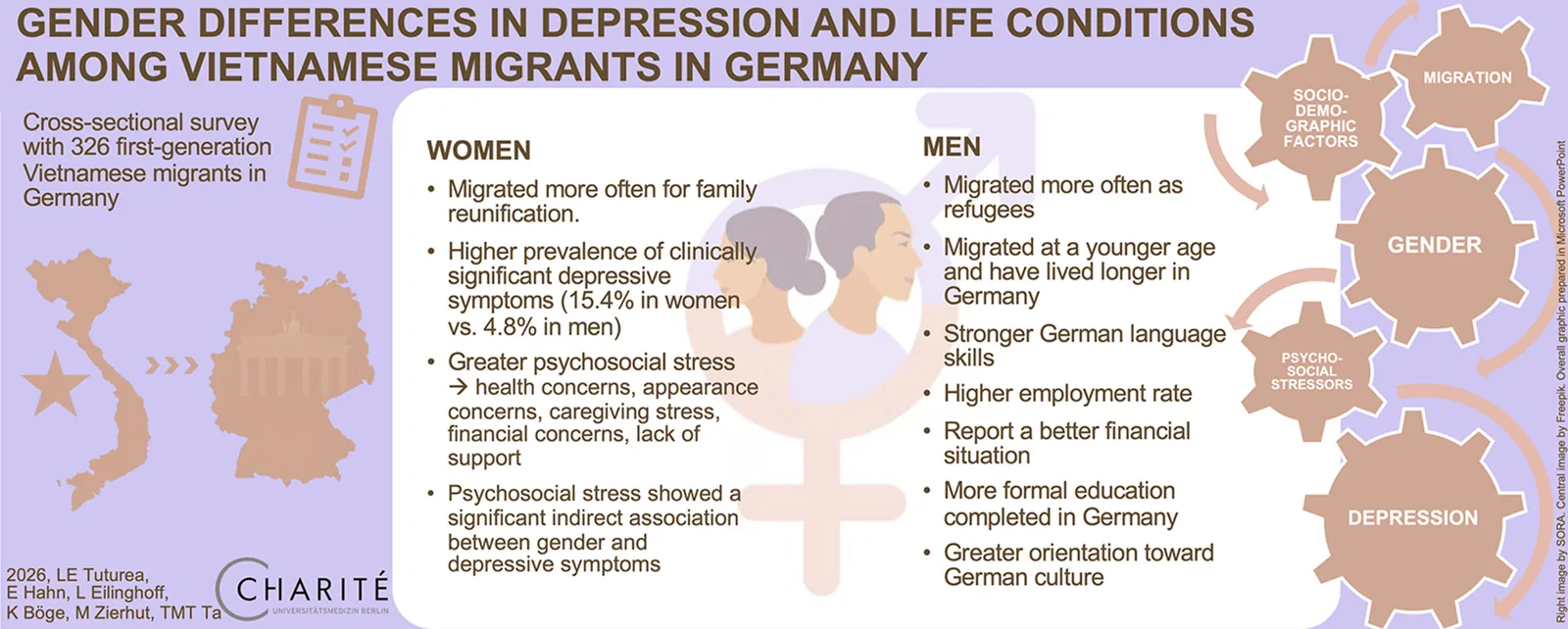

## 1 Exploring gender differences in depressive symptoms and life conditions among first-generation Vietnamese migrants in Germany

Women^1^ constitute roughly half of the world’s 272 million migrants, relocating for multiple reasons such as work, education, family reunification, or safety (UN Women, 2025). Their contributions are vital, spanning the introduction of new ideas, meeting labor demands, caregiving, remittance flows, and the enrichment of local communities. Yet female migrants often face compounded disadvantages rooted in structural inequality and cultural expectations (UN Women, 2025). This “double burden” of gender and migrant status is illustrated by the common pattern in which men migrate first and quickly enter the labor market, whereas women follow their spouses, arrive later, and assume primary childcare responsibilities; as a result, they frequently have lower language proficiency and reduced employment prospects compared with foreign-born men and native‑born women (OECD, 2020).

Migration entails not only geographic relocation but also far‑reaching sociocultural and psychological adjustments that can impact mental health (Gleeson et al., 2020; McAuliffe and Oucho, 2024). Emerging evidence shows that these adjustment stresses interact with gender‑related factors—such as cultural expectations, structural inequalities, and unequal access to coping resources—producing distinct patterns of mental‑health risks among migrant women and men (Alegría et al., 2017; Bornstein, 2017; Leu et al., 2011; Rommel et al., 2015; Choy et al., 2021). For example, a nationally representative psychiatric survey of Asian immigrants in the United States showed that family conflict exerted a stronger influence on psychological distress among women who maintained a strong connection to their ethnic culture (Leu et al., 2011). In contrast, for men, routine experiences of discrimination were more closely tied to poorer mental health outcomes when their English proficiency was limited (Leu et al., 2011). Because migration policies rarely reflect gender-specific experiences, collecting gender‑disaggregated data is essential to promote equality and enhance community well‑being (WHO, 2022; McAuliffe and Oucho, 2024; UN Women, 2025).

### 1.1 Gender gap in depression

Women worldwide are on average twice as likely to report depressive symptoms as men (Girgus and Yang, 2015; Kuehner, 2017; Salk et al., 2017; Hyde and Mezulis, 2020; WHO, 2022). International research shows that this ratio holds true across various nations and cultures (Kuehner, 2017; Hyde and Mezulis, 2020). This gender depression gap is often attributed to biological differences as well as structural and social inequalities (Girgus and Yang, 2015; Kuehner, 2017). On the other hand, gender and culture also impact the expression of depressive symptoms (Ryder et al., 2008; Gutierrez-Rojas et al., 2020; Otten et al., 2021; Shi et al., 2021). Men's adherence to traditional masculine norms may result in underreporting of symptoms and diminished help-seeking behavior (Otten et al., 2021; Shi et al., 2021). An atypical symptom profile, characterized by irritability and substance abuse, can result in under-detection (Otten et al., 2021; Shi et al., 2021). Such under-detection is particularly concerning given the strong link between severe depression and suicidal ideation in men, who accounted for 73% of recorded suicides in Germany in 2023 (Statistisches Bundesamt, 2025), underscoring the significance of gender-sensitive approaches (Otten et al., 2021; Shi et al., 2021).

To account for gender disparities in depression, many reviews group potential explanations into biological factors such as genetics and hormones, sociodemographic factors like employment and education, and psychosocial factors including gender roles and the burden of care work, experiences of violence and coping strategies (Girgus and Yang, 2015; Kuehner, 2017; Salk et al., 2017; Hyde and Mezulis, 2020). Recent models emphasize that gender differences in depression arise from dynamic interactions between multiple factors rather than a single cause (Girgus and Yang, 2015; WHO, 2022). Girgus and Yang (2015) propose three models linking gender to depression. First, the diathesis–stress model suggests that pre-existing vulnerabilities, such as hormonal fluctuations, interact with external stressors like relationship conflicts to trigger depressive symptoms. Second, the mediation model assumes that stressors activate vulnerabilities that then lead to depression; for example, lack of social support may function as a mediator linking gender to depressive outcomes. Finally, the transactional or stress–generation model emphasizes a bidirectional process whereby depressive symptoms themselves can give rise to additional stressors, such as relationship conflicts, which in turn reinforce the depressive state (Girgus and Yang, 2015). To address mental health disparities also from a sociological framework, Crenshaw’s (1989) intersectionality theory underscores that gender must be considered alongside other dimensions of inequality, such as ethnicity, education and financial status, which is particularly relevant in the context of migration, where these dimensions often converge.

In an international meta-analysis of migrant populations, approximately one in four individuals was found to experience depression (Bedaso and Duko, 2022). These prevalences were higher than usually found in general population samples. At the same time, they varied across migration types such as refugees or migrant workers, highlighting both the general burden of migration and the role of migration type. Within this review, female gender emerged as the most frequently reported sociodemographic correlate of depression, with women showing approximately two-and-a-half times higher likelihood of experiencing depression compared to men, while non-partnered marital status and low social support were also identified as significant risk factors (Bedaso and Duko, 2022). In Germany, the gender gap also differs across migrant subgroups: among refugees, women had about twice the prevalence of severe psychological distress compared to men (2.0:1; Walther et al., 2020), whereas among migrants with predominantly permanent residency, the gap in depression was smaller (1.3:1; Bartig et al., 2023). Large-scale cohort data further show that depressive symptoms follow an educational gradient, amplified by gender and migration, leaving migrant women at the highest risk (Vonneilich et al., 2025).

Thus, while gender gaps in mental health among migrants in Germany are evident, specific patterns differ across studies. Previous research has rarely examined mediation effects, overlooking how gender-related factors may indirectly shape outcomes (Otten et al., 2021). Overall, mechanisms remain largely unexplored, as findings are not transculturally generalizable and various sociodemographic and migration-related variables (e.g., residence status, language proficiency, cultural orientation, discrimination) as well as psychosocial stressors (e.g., caregiving burdens, lack of social support, experience of traumatic events) interact in complex ways (Leu et al., 2011; Rommel et al., 2015; Alegría et al., 2017; Bornstein, 2017; Otten et al., 2021; Bedaso and Duko, 2022; Bartig et al., 2023).

### 1.2 Vietnamese migrants in Germany

Germany hosts 14 million migrants, making it the second largest recipient country worldwide (McAuliffe and Oucho, 2024). About 25 million people in Germany are either immigrants or their children, representing roughly one-third of the population (Statistisches Bundesamt, 2023). The term first-generation migrant refers to individuals born outside Germany who migrated there during their lifetime (Rommel et al., 2015). Among Germany’s Southeast Asian migrant communities, the Vietnamese are the predominant group, numbering around 215,000 (Statistisches Bundesamt, 2023). Vietnamese migration to Germany has occurred in several distinct waves, including refugee, labor, and family-related migration, resulting in distinct opportunities and stressors (Ha, 2020). Existing research has identified depression as the most frequently diagnosed mental health disorder among Vietnamese migrants in Germany (Ta et al., 2014). Studies have reported that first-generation Vietnamese migrants exhibit depressive symptoms at rates comparable to those of the native-born Germans (Hahn et al., 2016). However, prior investigations have primarily relied on clinical samples, which may not accurately represent the overall Vietnamese migrant community. Regarding gender disparities, Tuturea et al. (2023) observed that first-generation Vietnamese men exhibit a stronger cultural orientation toward German society compared to their female counterparts, and that this higher orientation correlates with lower levels of depression. These findings suggest that gender-specific factors may play an important role in shaping mental health outcomes in this population.

### 1.3 Aim of the study

This study investigates the interplay of gender-specific experiences with sociodemographic, migration-related, and psychosocial factors. To improve generalizability and to examine gendered life conditions alongside depressive symptoms, this study targets a nonclinical community sample. This comprehensive approach will document gender differences and explore the underlying mechanisms driving these disparities, with the aim of informing targeted and effective policies and interventions for Vietnamese migrants in Germany.

We expected that men would report higher employment, language proficiency, and orientation toward German culture, whereas women would report more psychosocial stressors. Moreover, we hypothesized that women would report higher levels of depressive symptoms. Additionally, we exploratively examined whether selected factors that differed by gender statistically mediated the association between gender and depressive symptoms.

## 2 Methods

### 2.1 Procedure

Eligible participants were adult first-generation Vietnamese migrants. Participants were recruited in 2017–2018 via email invitations and personal outreach disseminated through the German Network for Mental Health for Vietnamese Migrants^2^ and its member organizations. A total of 365 individuals participated in the study and received a €5 compensation. Participants provided written informed consent, agreeing to the use of their data for research purposes. Ethical approval was granted by the Charité Universitätsmedizin Berlin ethics committee (reference EA2/116/15).

### 2.2 Key variables

Key variables were selected based on comprehensive review articles addressing factors influencing the relationship between gender differences and depressive symptoms (Piccinelli and Wilkinson, 2000; Girgus and Yang, 2015) as well as those between migration and depression (Sam and Berry, 2006; Leu et al., 2011; Alegría et al., 2017; Bornstein, 2017; Gleeson et al., 2020; Choy et al., 2021; Bartig et al., 2023; Vonneilich et al., 2025). This process yielded the following factors: sociodemographic variables included age (years), marital status (having a partner vs. not having a partner), children (yes/no), occupational status (working vs. not working), financial status (not sufficient, moderate, sufficient), and educational status in Germany and Vietnam (no education, education completed, studies completed). Migration-related variables included German language proficiency (none, few, moderate, good, very good), reason for migration (family, work/education, refugee), residency status (secure, e.g., German citizenship, permanent or long-term residence permit; insecure, e.g., temporary, limited, pending asylum status, or no residence permit), discrimination experiences (yes/no), religiosity (none, low, moderate, high), age at migration, years in Germany, and cultural orientation. Psychosocial factors encompassed health concerns, appearance concerns, lack of sexual desire/enjoyment, relationship conflicts, family caregiving stress, occupational stress, financial concerns, lack of social support, recent adverse event, and past traumatic memories (and dreams).

### 2.3 Measures

Data collection involved a structured Vietnamese-language survey assessing sociodemographic variables, migration-related aspects, and clinical factors (Ta et al., 2014).

Orientation toward German and Vietnamese culture was measured with the Stephenson Multigroup Acculturation Scale (SMAS; Stephenson, 2000), a 32-item Likert-scale assessing cultural practices such as language and media use. The host (15 items; range 15–60) and ethnic (17 items; range 17–68) subscales yield sum scores, with higher scores indicating stronger orientation. Meta-analytic data indicate that the SMAS is one of the most widely used acculturation measures and shows good reliability (Huynh et al., 2009).

Depressive symptoms and psychosocial stressors were assessed with two distinct modules of the Patient Health Questionnaire (PHQ; Kroenke et al., 2001; Löwe et al., 2002). The PHQ-9 covers nine DSM-IV depression criteria (range: 0–27) using a four-point response scale from not at all (0) to nearly every day (3), with scores ≥10 indicating clinically significant depressive symptoms (Manea et al., 2015). PHQ-9 total scores were calculated for participants who completed at least seven of the nine items; when one or two items were missing, the respondent-specific mean of the completed items was prorated to the nine-item scale. The PHQ Psychosocial Stress module (Löwe et al., 2002) consists of a checklist of ten single-item psychosocial stressors assessing health concerns, appearance concerns, lack of sexual desire/enjoyment, relationship issues, family caregiving stress, occupational stress, financial concerns, lack of support, traumatic memories, and recent adverse event. Each item is rated as not bothered (0), bothered a little (1), or bothered a lot (2). Following the PHQ manual, the item scores are summed to yield an overall Psychosocial Stress score ranging from 0 to 20. Complete responses to all ten items were required to calculate the total score. As no validated Vietnamese versions existed, German versions were translated via back-translation.

### 2.4 Statistical analysis

All analyses were carried out using SPSS version 29 and R version 4.4.2. To evaluate factorial validity, one-factor CFAs were conducted for the translated PHQ-9 and PHQ Psychosocial Stress items and estimated with WLSMV to account for the ordinal response format. To assess the empirical distinguishability of depressive symptoms and Psychosocial Stress, a combined one-factor model, in which all 19 items loaded on a common factor, was compared with a correlated two-factor model, in which the PHQ-9 and PHQ Psychosocial Stress items loaded on separate factors. The models were estimated with WLSMV using pairwise available observations and compared using a scaled chi-square difference test. Absolute model fit was evaluated using scaled and robust CFI, TLI, and RMSEA, together with SRMR, and the latent correlation between the two factors was examined. Additional construct validity checks for the translated PHQ-9 and PHQ Psychosocial Stress were conducted using Spearman correlations with related symptom measures.

Descriptive statistics were used to summarize the sample characteristics. Group differences were tested using *t*-tests or nonparametric equivalents, chi-square for categorical variables, and correlations (Spearman) for associations. Gender differences in magnitude of these correlations were evaluated using a permutation test with 1000 iterations. Significance for group differences and correlations was set at *p* ≤ .05 (Benjamini–Hochberg corrected).

Finally, exploratory Structural Equation Modeling (SEM) was employed to examine the mediating role of key variables in the relationship between gender and depressive symptoms. A sensitivity analysis using R semPower (v2.1.0) indicated that, for the specified SEM (*df* = 22) with the final sample size of *N* = 326, α = .05, and power = .80, the minimally detectable effect was *F₀* = 0.067, equivalent to Root Mean Square Error of Approximation (RMSEA) = 0.055, corresponding to a small-to-moderate effect size. A weighted least squares estimator with mean and variance adjustment (WLSMV) was used for model estimation. Missing data were handled using pairwise deletion and robust standard errors and confidence intervals were reported for the parameter estimates. In the mediation model, variables were chosen based on observed gender differences in key variables in the previous step. The observed PHQ Psychosocial Stress total score was included as one of the mediators in the structural equation model.

As a supplementary analysis, a twofold Oaxaca–Blinder decomposition of continuous PHQ-9 scores was conducted using the same predictors as the retained mediation model to distinguish the components associated with group differences in characteristics and estimated coefficients. Missing covariate data were multiply imputed specifically for this analysis; full methodological details and robustness checks are reported in Supplement Table 6.

### 2.5 Drop-Out analysis

Of the initial 365 participants, 23 were excluded for not providing key demographic information (gender or place of birth Vietnam), and an additional 16 participants were excluded due to having >20% missing data, resulting in a final sample of *N* = 326.

## 3 Results

### 3.1 Psychometric properties

All measures showed good to excellent internal consistency (SMAS German cultural orientation, α = .86; SMAS Vietnamese cultural orientation, α = .82; PHQ-9, α = .90; and PHQ Psychosocial Stress, α = .87). In the combined CFA conducted to examine discriminant validity between depressive symptoms and Psychosocial Stress, the correlated two-factor model fit significantly better than the one-factor model, scaled Δχ²(1) = 56.02, *p* < .001. Absolute fit of the two-factor model was acceptable to good according to the scaled indices, whereas the robust indices were less favorable. The latent correlation between depressive symptoms and Psychosocial Stress was high, *r* = .83, 95% CI [.78, .87], *p* < .001. The one-factor CFA of the PHQ-9, estimated with WLSMV for ordinal indicators, showed acceptable-to-good model fit according to the scaled indices, although the robust indices were less favorable. Standardized factor loadings ranged from .760 to .904. The one-factor CFA of the PHQ Psychosocial Stress items also showed acceptable-to-good model fit according to the scaled indices, although robust indices were less favorable. Standardized factor loadings ranged from .604 to .876. Full model fit indices for all four CFA models are presented in Supplement Table 1. Additional construct validity checks showed positive associations with related symptom measures, broadly consistent with prior psychometric findings for the PHQ-9 (Kliem et al., 2024) and PHQ Psychosocial Stress (Petrowski et al., 2024). Full correlation results and their psychometric context are presented in Supplement Table 2.

### 3.2 Sample characteristics

Sample characteristics are presented in Table 1. The final sample comprised N = 326 participants (39.0% men, 61.0% women) with a mean age of 44.8 years (SD = 12.4).

**Table 1:** Sample characteristics and gender differences.

	**Overall**		**Men**	**Women**	**Missing values**	**Gender Differences**		
**Variable**	***n***	***%, M(SD)***	***%, M(SD)***	***%, M(SD)***	**Overall (Men, Women)**	**Statistic**	***p***	**Effect Size**
Gender	326		38.96%	61.04%				
**Depressive symptoms**	321	3.71 (4.34)	**2.79 (3.33)**	**4.31 (4.79)**	1.53% (0.79%, 2.01%)	U = 10218.50, z = −2.58	.019	*r* = 0.14
Age (years)	304	44.84 (12.44)	45.36 (13.06)	44.5 (12.03)	6.75% (4.72%, 8.04%)	t(302) = 0.58	.628	*d* = 0.07
Marital status (has partner)	319	55.17%	59.20%	52.58%	2.15% (1.57%, 2.51%)	χ²(1) = 1.09	.355	φ = 0.06
Children (yes)	285	85.61%	81.08%	88.51%	12.58% (12.6%, 12.56%)	χ²(1) = 2.46	.191	φ = 0.09
**Occupational status (working)**	301	69.10%	**78.99%**	**62.64%**	7.67% (6.3%, 8.54%)	χ²(1) = 8.26	.012	φ = 0.17
**Financial status**	301				7.67% (3.15%, 10.55%)	U = 13278.50, z = 3.44	.003	*r* = 0.2
Not sufficient	60	19.93%	12.20%	25.28%				
Moderate	155	51.50%	50.41%	52.25%				
Sufficient	86	28.57%	37.4%	22.47%				
**Education Germany**	272				16.56% (12.6%, 19.1%)	U = 10318.00, z = 2.57	.019	*r* = 0.16
No education	178	65.44%	55.86%	72.05%				
Education completed	53	19.49%	26.13%	14.91%				
Studies completed	41	15.07%	18.02%	13.04%				
Education Vietnam	321				1.53% (2.36%, 1.01%)	U = 11381.50, z = −1.11	.355	*r* = 0.06
No education	142	44.24%	45.97%	43.15%				
Education completed	111	34.58%	37.90%	32.49%				
Studies completed	68	21.18%	16.13%	24.37%				
**Age at migration**	299	24.79 (8.36)	**23.09 (7.55)**	**25.93 (8.7)**	8.28% (5.51%, 10.05%)	*t*(297) = −2.91	.012	*d* = 0.34
**Years in Germany**	312	20.06 (11.58)	**22.34 (12.10)**	**18.55 (10.99)**	4.29% (2.36%, 5.53%)	*t*(310) = 2.86	.012	*d* = 0.33
**Reason for Migration**	306				6.13% (2.36%, 8.54%)	χ²(2) = 16.49	.003	*V* = 0.23
Family	77	25.16%	**15.32%**	**31.87%**		χ²(1) = 9.86	.005	φ = 0.18
Work/Education	193	63.07%	66.13%	60.99%		χ²(1) = 0.63	.427	φ = 0.05
Refugee	36	11.76%	**18.55%**	**7.14%**		χ²(1) = 8.18	.006	φ = 0.16
Residency status (secure)	320	65.62%	67.46%	64.43%	1.84% (0.79%, 2.51%)	χ²(1) = 0.19	.662	φ = 0.02
**German language proficiency**	313				3.99% (1.57%, 5.53%)	*U* = 9464.00, *z* = −3.04	.011	*r* = 0.17
Very good	60	19.17%	24.00%	15.96%				
Good	40	12.78%	16.00%	10.64%				
Moderate	119	38.02%	39.20%	37.23%				
Few	83	26.52%	17.60%	32.45%				
None	11	3.51%	3.20%	3.72%				
**German cultural orientation**	326	39.2 (8.32)	**41.27 (7.85)**	**37.88 (8.36)**	0% (0%, 0%)	*t*(324) = 3.65	.003	*d* = 0.41
Vietnamese cultural orientation	326	58.34 (6.31)	58.57 (7.02)	58.19 (5.82)	0% (0%, 0%)	*t*(324) = 0.54	.628	*d* = 0.06
Discrimination experiences	317	22.71%	19.20%	25.00%	2.76% (1.57%, 3.52%)	χ²(1) = 1.14	.355	φ = 0.06
Religiosity	303				7.06% (3.94%, 9.05%)	*U* = 10014.00, *z* = −1.45	.219	*r* = 0.08
None	126	41.58%	46.72%	38.12%				
Low	38	12.54%	13.11%	12.15%				
Moderate	95	31.35%	26.23%	34.81%				
High	44	14.52%	13.93%	14.92%				
**Psychosocial Stress**	295	4.26 (4.18)	**3.32 (3.15)**	**4.87 (4.65)**	9.51% (7.87%, 10.55 %)	*U* = 8596.50, *z* = −2.55	.019	*r* = 0.15
*Note. N* = 326. Values are reported as means (and standard deviations) for continuous variables or percentages for categorical variables. Gender differences were analyzed using t tests (Cohen’s *d*: 0.2 = small, 0.5 = medium, 0.8 = large), Mann–Whitney U tests (*r*: 0.10 = small, 0.24 = medium, 0.37 = large), or chi-square tests (φ/*V*: 0.1 = small, 0.3 = medium, 0.5 = large). Mann–Whitney *z* values include tie and continuity corrections; *r* uses tie correction only. All tests were two-tailed, with *p* < .05 as the criterion for statistical significance. *p* values were Benjamini–Hochberg-adjusted across 18 main tests and separately across three migration-reason post-hoc tests. Boldface indicates significant gender differences.								

### 3.3 Gender differences in key variables

Gender differences across key variables are reported in Table 1. Overall, 15.4% of females and 4.8% of males met the PHQ-9 cutoff for clinically significant depressive symptoms (PHQ-9 ≥ 10). Consistent with the hypothesis, females had significantly higher odds of meeting this cutoff (*OR* = 3.64, 95% *CI* [1.6, 9.9]) and reported significantly higher depression scores and higher levels of psychosocial stress than men. Also consistent with the hypothesis, a significantly larger proportion of men were employed, had completed more formal education in Germany, and reported a better financial situation, migrated at a younger age, spent significantly more years in Germany, demonstrated better German language proficiency, and showed a stronger orientation toward German culture than women. Men were more likely to migrate as refugees, whereas women were more likely to migrate for family reunification. Effect sizes for these significant group differences ranged from small to borderline medium. No significant gender differences were found for age, marital status, having children, education in Vietnam, migrating for work or educational reasons, residency status, Vietnamese cultural orientation, perceived discrimination, or religiosity.

Further examination of psychological stressor items revealed that women experienced significantly higher levels of health concerns, appearance concerns, family caregiving stress, financial concerns, and a perceived lack of support than men, with effect sizes in the small range. By contrast, there were no significant gender differences for lack of sexual desire/enjoyment, relationship issues, occupational stress, past traumatic memories, or recent adverse event (see Supplement Table 3 for complete details).

### 3.4 Associations between depressive symptoms and key variables

The correlation analyses for the relationships between depressive symptoms and key variables can be found in Table 2. Depressive symptoms were significantly associated with occupational status, financial status, German language proficiency, discrimination experiences, German cultural orientation, Vietnamese cultural orientation, and psychosocial stress. Notably, Vietnamese cultural orientation showed a significant negative correlation with depressive symptoms among men but not among women; the between-gender difference was significant before, but not after the Benjamini-Hochberg correction. Supplement Table 4 further details that all individual psychosocial stressor items were positively correlated with depressive symptoms. Before applying the Benjamini-Hochberg correction, women showed significantly stronger correlations than men between depressive symptoms and lack of sexual desire/enjoyment, perceived lack of support, and recent adverse events. After applying the Benjamini–Hochberg correction, only the gender difference for recent adverse events remained significant.

**Table 2:** Correlations of depressive symptoms and key variables.

**Variable**	**Overall**	**Men**	**Women**	**Difference**
Age (years)	.00	-.02	.03	.05
Marital status (has partner)	-.07	-.02	-.08	-.06
Children (yes)	-.11	-.13	-.13	.01
Occupational status (working)	-.15**	-.23(*)	-.09	.14
Education Germany	-.08	-.03	-.07	-.03
Education Vietnam	-.01	.11	-.09	-.20
Financial status	-.16**	-.25**	-.07	.19
Age at migration	.08	-.05	.11	.16
Years in Germany	-.10	-.01	-.11	-.10
Residency status (secure)	-.09	.04	-.16(*)	-.20
German language proficiency	.17**	.09	.19**	.10
Religiosity	-.06	.01	-.13	-.14
Discrimination experiences	.23**	.21(*)	.23**	.02
German cultural orientation	-.17**	-.11	-.17(*)	-.07
Vietnamese cultural orientation	-.15**	-.32**	-.02	.30(**)
Psychosocial Stress	.67**	.66**	.68**	.02

*Note. N* = 326. Pairwise sample sizes vary because correlations were calculated using complete observations for each variable pair. Spearman rank correlations. Difference values represent women’s correlation minus men’s correlation. The permutation test was used to compute group differences with 1000 permutations. *p < .05; **p < .01. Asterisks in parentheses indicate that the result is no longer significant after applying the Benjamini–Hochberg correction for multiple comparisons.

### 3.5 Gender differences in depressive symptoms

#### 3.5.1 Mediation model

Fig. 1 presents the mediation of gender differences in depressive symptoms through key variables and displays standardized estimates β and associated *p*-values. Unstandardized estimates, the robust 95% confidence intervals, and associated *p*-values are provided in Supplement Table 5. The SEM showed acceptable-to-good model fit: χ²(22) = 47.05, *p* = .001, CFI = 0.955, TLI = 0.888, RMSEA = 0.059 [0.036, 0.083], SRMR = 0.059. Overall, the model explained 58.3% of the variance in depressive symptoms, which represents a large effect (Cohen, 1988). Although the direct effect of gender on depressive symptoms was not significant, the total effect, which reflects both direct and indirect pathways, was significant. The significant indirect effect via psychosocial stress, with other indirect paths not reaching significance, indicates that psychosocial stress was the only significant indirect pathway in this cross-sectional model.

**Fig. 1:**
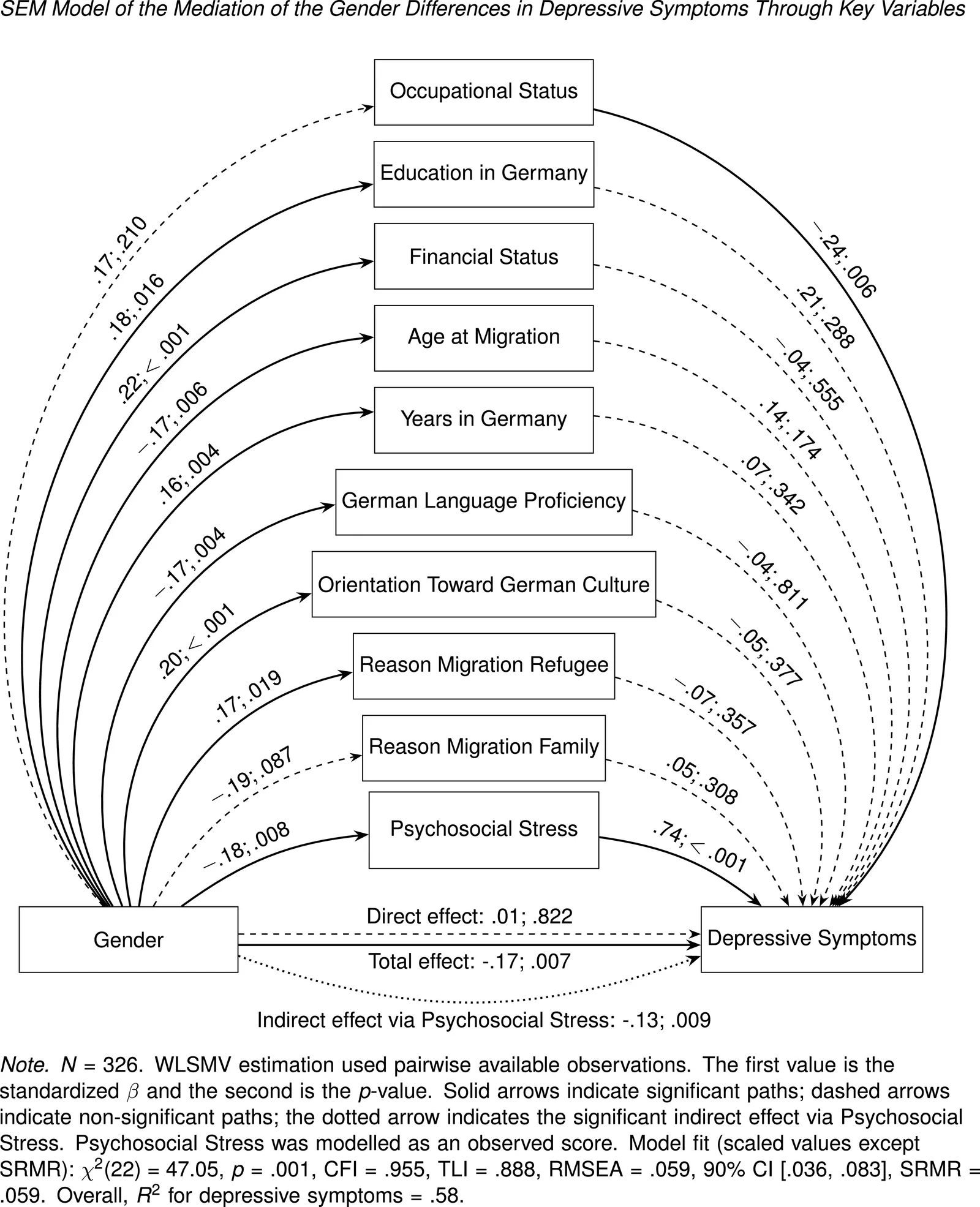
SEM Model of the Mediation of the Gender Differences in Depressive Symptoms Through Key Variables. Indirect effect via Psychosocial Stress:−.13; .009 Note. N = 326. WLSMV estimation used pairwise available observations. The first value is the standardized β and the second is the *p*-value. Solid arrows indicate significant paths; dashed arrows indicate non-significant paths; the dotted arrow indicates the significant indirect effect via Psychosocial Stress. Psychosocial Stress was modelled as an observed score. Model fit (scaled values except SRMR): χ2(22) = 47.05, *p* = .001, CFI = .955, TLI = .888, RMSEA = .059, 90% CI [.036, .083], SRMR = .059. Overall, R2 for depressive symptoms = .58.

### 3.6 Supplementary Oaxaca–Blinder decomposition

In the supplementary Oaxaca–Blinder decomposition, the observed mean difference was 1.51 PHQ-9 points (95% CI [0.63, 2.40]). The characteristics component was 1.47 points (95% CI [0.69, 2.24]; 97.0%), whereas the coefficients component was 0.05 points (95% CI [−0.69, 0.78]). This pattern was robust across alternative reference structures, and psychosocial stress showed the largest individual contribution (see Supplement Table 6 for complete details).

## 4 Discussion

### 4.1 Main findings

This study is the first to explore how gender influences life conditions and depressive symptoms in a large community sample of first-generation Vietnamese migrants in Germany, revealing pronounced differences between men and women. Women reported significantly higher depression scores compared to men, with 3.64-fold greater odds of meeting the PHQ-9 cutoff for clinically significant depressive symptoms. Men migrated at a younger age, lived longer in Germany, and showed higher language proficiency and cultural orientation. Furthermore, men were more likely to be employed, had completed more formal education in Germany, and reported a better financial situation. In terms of migration motivations, while most participants migrated for occupational or educational purposes across genders, men more frequently migrated as refugees, whereas women more frequently migrated for family reunification. Additionally, women reported more psychosocial stressors, including health concerns, appearance concerns, family caregiving responsibilities, financial concerns, and a lack of support. Correlation analyses further indicated a trend whereby both genders exhibited positive associations between depressive symptoms and low financial status, discrimination experiences, low German language proficiency, and low German cultural orientation, as well as with measured psychosocial stressors (health concerns, appearance concerns, lack of sexual desire/enjoyment, relationship issues, family caregiving stress, occupational stress, financial concerns, lack of support, past traumatic memories, recent adverse event). Interestingly, the relationships between depressive symptoms and diminished sexual desire/enjoyment, perceived lack of support, and adverse events tended to be more pronounced among women, while a stronger orientation toward Vietnamese culture was associated with fewer depressive symptoms among men. However, after controlling for multiple testing using the Benjamini–Hochberg procedure, most of these between-gender differences no longer reached significance and should therefore be regarded as exploratory. The mediation analysis revealed that while these socioeconomic and migration-related factors significantly differed by gender, none of these variables showed a significant indirect effect linking gender to depressive symptoms. In contrast, psychosocial stress showed a significant statistical indirect association between gender and depressive symptoms in the cross-sectional model. The supplementary Oaxaca–Blinder decomposition yielded a complementary descriptive result: the characteristics component corresponded to 97.0% of the observed PHQ-9 difference, with psychosocial stress making the largest individual contribution. The aggregate coefficients component was close to zero but was estimated with considerable uncertainty and should not be interpreted as evidence that associations were identical across genders.

### 4.2 Gender differences in socioeconomic and migration-related factors

In the general German population, gender differences in formal qualifications are small and employment gaps have narrowed; nevertheless, women remain less often employed, more frequently work part-time, and earn less, partly reflecting family responsibilities and limited advancement opportunities (Statistik der Bundesagentur für Arbeit, 2024).

Migrant women in this study also reported a higher burden of caregiving and limited career and economic advancement, poorer financial situation and heightened financial concerns compared to their male counterparts. Although educational levels were comparable in the country of origin, this parity did not translate into equal opportunities after migration, as their overall labor force participation and education status in Germany were lower than those of migrant men. These accentuated patterns of structural gender inequality for migrant women are consistent with decades of prior migration research (Sam and Berry, 2006; OECD, 2020). In Germany, large-scale panel data on refugees demonstrate limited labor market access, educational–occupational mismatches, lower wages, and a higher risk of poverty among migrant women compared to men (Brücker et al., 2019; Lechner et al., 2023). Crucially, qualifications in regulated professions often chosen by women, such as nursing and educations are frequently not recognized (Lechner et al., 2023). At the same time, women participate less frequently in language courses, integration measures, and vocational training, and report fewer social contacts with native Germans (Brücker et al., 2019; Walther et al., 2020; Lechner et al., 2023). Interestingly, newly arrived female migrants in Germany view migration as an opportunity to take on more active roles in education, employment, and family relationships (Walther et al., 2021). Thus, a lower orientation toward German culture may not reflect a lack of interest but rather constrained opportunities to interact outside the family sphere (Huynh et al., 2009). Longitudinal data show that migrant mothers’ employment is strongly tied to daycare availability, while fathers remain unaffected (Brücker et al., 2023). Women’s predominant responsibility for household and caregiving work is likewise well documented among Vietnamese women, both in Vietnam and within Vietnamese diaspora communities (Nguyen et al., 2021; Do et al., 2023). Besides traditional gender role divisions, another underlying mechanism could be that, if a woman migrates later for family reunification, as reflected in our data, her husband has often already built social networks, acquired language skills, and gained work experience in Germany, which makes it more practical for the woman to focus on domestic responsibilities (Lechner et al., 2023). Such migration-specific barriers can provide the backdrop for understanding why depressive symptoms and psychosocial stressors are distributed unevenly across genders in our sample.

### 4.3 Gender differences in depression among Vietnamese migrants

Globally, meta-analytic data from 30 countries indicate an aggregate depression prevalence of 14.4% for women and 11.5% for men (Lim et al., 2018). German representative data show a smaller gap (9.8% vs. 8.5%; PHQ-8 ≥ 10; Hapke et al., 2022), whereas migrants report much higher rates (23.6% vs. 18.0%; PHQ-9 ≥ 10; Bartig et al., 2023). In our sample, prevalence was intermediate (15.4% vs. 4.8%). This corresponds to a prevalence ratio of about 3:1, while the odds ratio was 3.64 (reflecting the different calculation methods), thus exceeding the commonly reported 2:1 gender gap (Girgus and Yang, 2015; Kuehner, 2017; Salk et al., 2017; WHO, 2022).

Taken together with the previously reported differences in sociodemographic and migration-related variables, our findings, along with other empirical evidence, suggest that female migrants may be at increased risk of social isolation (Sam and Berry, 2006). Qualitative findings reveal that female Vietnamese migrants in Germany express distress over their partners' lack of involvement in household chores (Nguyen et al., 2021). Women tended to show stronger associations between depressive symptoms and psychosocial stressors such as lack of support, recent adverse event, and lack of sexual desire/enjoyment; after Benjamini–Hochberg correction, only the gender difference for recent adverse events remained significant. In contrast, before applying the Benjamini–Hochberg correction, occupational and financial status were significantly correlated with depressive symptoms among men but not women; after correction, only the correlation with financial status among men remained significant. Indeed, the underlying mechanisms might be more complex. German survey data point to heightened psychological distress among women with low participation in educational programs (Walther et al., 2020). Furthermore, while paid work generally reduces the prevalence of depressive disorders in both genders, its benefits for women appear restricted to those without children, suggesting that role overload may offset potential mental health gains (Piccinelli and Wilkinson, 2000).

Surprisingly, although both Vietnamese men and women exhibited a high orientation toward their heritage culture, this study’s findings suggest that only for men this orientation might be associated with reduced depressive symptoms. One possible explanation could be that, since expectations of occupational success are primarily directed at men, belonging to the ethnic community might serve as an alternative source of recognition and may help them compensate for migration-related status loss (Kuehner, 2017; Walther et al., 2020). Role conflicts from traditional gender norms may limit the benefits of ethnic cultural orientation for migrated women (Piccinelli and Wilkinson, 2000; Leu et al., 2011; Bornstein, 2017), but further research is needed to investigate whether these findings are replicable and to explore potential underlying mechanisms.

### 4.4 Limitations and future directions

Several limitations must be considered when interpreting the results of this study. Given the cross-sectional design, neither the mediation analysis nor the Oaxaca–Blinder decomposition permits causal conclusions. While gender is often conceptualized as a causal factor, the status of other variables remains ambiguous in this cross-sectional design. Depressive symptoms may hinder proactive behaviors, including occupational engagement, and shape biased perceptions of factors such as health concerns, language proficiency, and experiences of discrimination. Consistent with this conceptual overlap, the high latent correlation between Psychosocial Stress and depressive symptoms warrants some caution in interpreting the indirect effect, although the significantly better fit of the two-factor model supports distinguishing the two constructs. Furthermore, among some Asian populations (Ryder et al., 2008; Gutierrez-Rojas et al., 2020), including Vietnamese (Dreher et al., 2017), health concerns may be understood as manifestations of depressive symptoms rather than as independent explanatory factors. It should also be noted that men may underreport depressive symptoms due to traditional masculine norms (Ryder et al., 2008; Gutierrez-Rojas et al., 2020; Otten et al., 2021; Shi et al., 2021), which could partly explain why we found lower prevalence rates among men than among women. Future studies should therefore consider gender-sensitive screening approaches.

Moreover, although this study demonstrated a moderate-to-good model fit, it is important to recognize that a substantial amount of variance in the mediation model remained unexplained and some effects observed in other studies could not be replicated. With a larger sample, some trends in the results might have approached significance. Additionally, the use of single-item questions may have impacted the reliability of some variables. In particular, analyses of individual psychosocial stressor items were based on single-item indicators and should therefore be interpreted cautiously and primarily as exploratory. Still, this approach was chosen to minimize participant burden. While various individual factors were examined, other potential contributors to the gender depression gap should be considered. For instance, sensitive factors such as gender-based violence should be measured appropriately. Also, factors such as biological differences and especially gender roles and identities might be particularly relevant, highlighting the need for more nuanced frameworks in future research (Kuehner, 2017; Hyde et al., 2019).

Leveraging its exploratory broad scope that screened many key concepts related to gender differences and migrant mental health, this study establishes a valuable foundation for more in-depth investigations. Future research should incorporate longitudinal designs (e.g., cross-lagged panel models) alongside comprehensive assessment tools and qualitative methods, to add deeper insights into these preliminary findings. Future studies with larger samples could use a Fairlie decomposition to examine the relative contributions of group differences in measured characteristics and estimated coefficients.

### 4.5 Implications

This study highlights the importance of addressing structural gender inequities and implementing culturally sensitive approaches to migrant women’s mental health. Recruitment in a nonclinical community setting makes the findings applicable beyond patient populations and relevant for targeted policy and participation measures, particularly by highlighting the significance of language fluency, financial situation, educational and job opportunities in the host culture, as well as reducing care burdens and addressing psychosocial stressors. Evidence suggests that reduced childcare responsibilities and the availability of specialized courses for migrant women can boost participation in integration programs, indicating that initiatives like language courses with accessible childcare may enhance societal participation and improve socioeconomic conditions (OECD, 2020; Brücker et al., 2023; Lechner et al., 2023). In addition, tailored counseling and employment support, improved access to financing of self-employment opportunities, as well as fostering civic engagement by supporting migrant self-organization and the development of social networks can further improve the situation for female migrants (Lechner et al., 2023). Studies have found that individuals who immerse themselves concurrently in both the host culture and their ethnic culture, tend to show overall more favorable outcomes, such as reduced depression (Choy et al., 2021; Tuturea et al., 2023).

Linguistic barriers may also limit equal access to the health care system (Lechner et al., 2023). Targeted health promotion, exemplified by the German Network for Mental Health for Vietnamese Migrants^2,^ can familiarize migrants with the local healthcare system and support building local networks (Lechner et al., 2023). The results suggest that culturally congruent medical and psychological care delivered in Vietnamese may benefit especially female Vietnamese migrants. More broadly, this study found positive associations between depressive symptoms and several psychosocial stressors, including health concerns, financial concerns, family caregiving stress, and perceived lack of social support. This highlights the need for training programs that equip health and social care workers with the skills to recognize particular migration-, cultural-, and gender-related stressors.

Finally, expanding health monitoring through more nuanced research on migrants would further improve the evidence base for needs assessments, policy, and resource allocation.

### 4.6 Conclusion

By integrating a more intersectional perspective, this study deepens the understanding of gendered migrant mental health and lays the groundwork for more effective policies and practices. Specifically, addressing psychosocial stressors while improving opportunities for societal participation may help enhance the living conditions of Vietnamese migrant women. Such measures include culturally sensitive services and counselling in the native language, accessible language courses and childcare, as well as facilitated entry to employment, which could in turn reduce financial strain. Overall, addressing structural gender inequities and integrating culturally sensitive approaches may help improve both the mental health and the broader life conditions of migrant women.

## Ethics declaration

Written informed consent to take part in the study and to publish the article has been obtained from all participants or their legal representatives. The privacy rights of participants have been observed.

This study was performed in compliance with relevant laws, regulatory frameworks and guidelines where the research took place. This study was approved by the Charité Universitätsmedizin Berlin ethics committee. (Approval No EA2/116/15).

## Declaration of generative AI and AI-assisted technologies in the writing process

During the preparation of this work, the authors used ChatGPT to improve the readability and language of the manuscript and to assist with developing and reviewing R code, checking statistical results, and refining their interpretation. After using this tool, the authors reviewed and edited the content as needed and take full responsibility for the content of the published article.

## Funding

This study was undertaken in part within the subproject D03 of the CRC 1171 “Affective Societies –Dynamics of Coexistence in Mobile Worlds” which received funding from the German Research Foundation (DFG – Deutsche Forschungsgemeinschaft).

## Data availability

Data are available from the corresponding and last author upon reasonable request.

## CRediT authorship contribution statement

**Laura Elisabeth Tuturea:** Writing – review & editing, Writing – original draft, Visualization, Validation, Software, Methodology, Formal analysis, Data curation, Conceptualization. **Eric Hahn:** Writing – review & editing, Supervision, Project administration, Investigation, Funding acquisition, Conceptualization, Methodology. **Luisa Eilinghoff:** Writing – review & editing, Visualization, Supervision, Conceptualization, Methodology. **Kerem Böge:** Writing – review & editing, Supervision, Project administration, Methodology, Funding acquisition, Conceptualization. **Marco Zierhut:** Writing – review & editing, Visualization, Conceptualization, Methodology. **Thi Minh Tam Ta:** Writing – review & editing, Supervision, Resources, Project administration, Methodology, Investigation, Funding acquisition, Data curation, Conceptualization.

## Declaration of competing interest

The authors declare that they have no known competing financial interests or personal relationships that could have appeared to influence the work reported in this paper.

## Footnotes

1 Consistent with most previous scientific literature, this paper employs a binary concept of gender as "male" and "female," without distinctly differentiating between biological sex and social gender (Hyde et al., 2019). However, the authors emphasize that future research should also explore a more nuanced conceptualization of gender.

2 https://www.netzwerk-vietpsygesundheit.de/de/.
