# Supplementary material for: Exploring gender differences in depressive symptoms and life conditions among first-generation Vietnamese migrants in Germany
Source: J Migr Health. 2026 Oct 15;14:100447. doi: 10.1016/j.jmh.2026.100447 (PMC13734991; doi:10.1016/j.jmh.2026.100447)
Supplement: Supplementary file 1 [file mmc1.pdf]

Supplement Table 1

Model Fit of the Separate and Combined Confirmatory Factor Analyses

Model	*N*	Scaled χ² (*df*), *p*	Scaled CFI	Robust CFI	Scaled TLI	Robust TLI	Scaled RMSEA [90% CI]	Robust RMSEA [90% CI]	SRMR
PHQ-9 one-factor CFA	323	75.46 (27), < .001	.989	.930	.986	.907	.075 [.055, .095]	.145 [.108, .184]	.044
PHQ Psychosocial Stress one-factor CFA	324	95.81 (35), < .001	.975	.900	.968	.871	.073 [.056, .091]	.144 [.110, .179]	.062
Combined 19-item one-factor CFA	326	498.43 (152), < .001	.953	.757	.947	.727	.084 [.076, .092]	.174 [.158, .190]	.084
Combined correlated two-factor CFA	326	315.78 (151), < .001	.978	.834	.975	.812	.058 [.049, .067]	.144 [.127, .162]	.064

Note. Models were estimated using weighted least squares mean- and variance-adjusted (WLSMV) estimation with pairwise available observations. Model-specific sample sizes are reported in the table. CFI = comparative fit index; TLI = Tucker–Lewis index; RMSEA = root mean square error of approximation; SRMR = standardized root mean square residual. The correlated two-factor model fit significantly better than the combined one-factor model, scaled $\Delta\chi^2(1) = 56.02$, $p < .001$. In the correlated two-factor model, the latent correlation was $r = .83$, 95% CI [.78, .87], $p < .001$.

Supplement Table 2

Construct Validity Checks for the PHQ-9 and PHQ Psychosocial Stress

Measure	Correlate	*n*	Spearman's rho	95% CI	BH-adjusted *p*
PHQ-9	BSI-18 Depression	319	.68	[.60, .75]	< .001
PHQ-9	BSI-18 Anxiety	319	.72	[.65, .77]	< .001
PHQ-9	BSI-18 Somatization	319	.66	[.59, .73]	< .001
PHQ-9	BSI-18 Global Severity Index	319	.77	[.71, .82]	< .001
PHQ Psychosocial Stress	PHQ-9	291	.67	[.60, .74]	< .001
PHQ Psychosocial Stress	BSI-18 Depression	292	.65	[.57, .72]	< .001
PHQ Psychosocial Stress	BSI-18 Anxiety	293	.68	[.60, .74]	< .001
PHQ Psychosocial Stress	BSI-18 Somatization	292	.62	[.54, .70]	< .001
PHQ Psychosocial Stress	BSI-18 Global Severity Index	292	.72	[.65, .78]	< .001

Note. PHQ = Patient Health Questionnaire; BSI-18 = Brief Symptom Inventory-18; CI = confidence interval; BH = Benjamini–Hochberg. Spearman correlations are reported with percentile bootstrap 95% confidence intervals based on 5000 bootstrap resamples. Sample sizes vary because correlations were calculated using pairwise complete observations.

Construct Validity Checks

For the PHQ-9, the selection and interpretation of construct validity correlates were guided by Kliem et al. (2024), a psychometric evaluation of the PHQ-9 in a large representative German general population sample. In that study, the PHQ-9 showed high positive correlations with the BSI-18 depression, anxiety, and somatization subscales and the BSI-18 Global Severity Index. Accordingly, positive associations between the translated PHQ-9 and these BSI-18 measures were expected in the present sample. As shown in Supplement Table 2, the translated PHQ-9 was strongly associated with BSI-18 depression, anxiety, somatization, and global severity scores. These correlations were positive, strong, and broadly comparable in magnitude to those reported by Kliem et al. (2024), supporting the expected pattern of convergent construct validity for the translated PHQ-9 in the present sample.

For the PHQ Psychosocial Stress, the selection and interpretation of construct validity correlates were guided by Petrowski et al. (2024), a psychometric evaluation of the PHQ Psychosocial Stress scale in a representative German general population sample. In that study, construct validity was examined using related symptom measures, including PHQ-9 depression, GAD-7 anxiety, and PHQ-15 somatic symptoms. The PHQ Psychosocial Stress scale showed moderate-to-strong positive correlations with these measures. Accordingly, positive associations between the translated PHQ Psychosocial Stress and the PHQ-9 as well as BSI-18 measures of depression, anxiety, somatization, and global severity scores were expected in the present sample. As shown in Supplement Table 2, PHQ Psychosocial Stress was moderately to strongly associated with the PHQ-9 and with BSI-18 depression, anxiety, somatization, and global severity scores. These findings were in line with expectations and support the expected pattern of convergent construct validity for the translated PHQ Psychosocial Stress in the present sample.

Supplement Table 3

Psychosocial Stressor Items and Gender Differences

		%			Missing Values	Gender Differences		
Variable	***n***	**Overall**	**Men**	**Women**	**Overall (Men, Women)**	**Statistic**	***p***	**Effect Size**
**Health Concerns**	324				0.61% (0.79%, 0.50%)	*U* = 10562.00, *z* = -2.55	.027	*r* = .14
Not Bothered	128	39.51%	46.03%	35.35%				
A Little Bothered	153	47.22%	46.83%	47.47%				
A Lot Bothered	43	13.27%	7.14%	17.17%				
**Appearance Concerns**	323				0.92% (0.79%, 1.01%)	*U* = 10084.50, *z* = -3.26	.010	*r* = .18
Not Bothered	191	59.13%	69.05%	52.79%				
A Little Bothered	106	32.82%	28.57%	35.53%				
A Lot Bothered	26	8.05%	2.38%	11.68%				
Lack of Sexual Desire/Enjoyment	310				4.91% (5.51%, 4.52%)	*U* = 10858.00, *z* = -0.89	.431	*r* = .05
Not Bothered	220	70.97%	73.33%	69.47%				
A Little Bothered	78	25.16%	25%	25.26%				
A Lot Bothered	12	3.87%	1.67%	5.26%				
Relationship Issues	313				3.99% (2.36%, 5.03%)	*U* = 11315.50, *z* = -0.67	.505	*r* = .04
Not Bothered	230	73.48%	75%	72.49%				
A Little Bothered	68	21.73%	22.58%	21.16%				
A Lot Bothered	15	4.79%	2.42%	6.35%				
**Family Caregiving Stress**	319				2.15% (0.79%, 3.02%)	*U* = 10352.00, *z* = -2.66	.026	*r* = .15
Not Bothered	207	64.89%	73.02%	59.59%				
A Little Bothered	82	25.71%	22.22%	27.98%				
A Lot Bothered	30	9.4%	4.76%	12.44%				
Occupational Stress	322				1.23% (0.79%, 1.51%)	*U* = 11080.50, *z* = -1.79	.118	*r* = .10
Not Bothered	192	59.63%	64.29%	56.63%				
A Little Bothered	107	33.23%	33.33%	33.16%				

Variable	*n*	% Overall	% Men	% Women	Missing Values Overall (Men, Women)	Gender Differences Statistic	*p*	Effect Size
A Lot Bothered	23	7.14%	2.38%	10.2%				
**Financial Concerns**	323				0.92% (0.79%, 1.01%)	U = 10111.50, z = -3.10	.010	r = .17
Not Bothered	130	40.25%	49.21%	34.52%				
A Little Bothered	157	48.61%	45.24%	50.76%				
A Lot Bothered	36	11.15%	5.56%	14.72%				
**Lack of Support**	321				1.53% (1.57%, 1.51%)	U = 10587.50, z = -2.44	.030	r = .14
Not Bothered	209	65.11%	72%	60.71%				
A Little Bothered	80	24.92%	24%	25.51%				
A Lot Bothered	32	9.97%	4%	13.78%				
Traumatic Memories	321				1.53% (1.57%, 1.51%)	U = 11301.00, z = -1.74	.118	r = .10
Not Bothered	262	81.62%	85.6%	79.08%				
A Little Bothered	39	12.15%	13.6%	11.22%				
A Lot Bothered	20	6.23%	0.8%	9.69%				
Recent Adverse Event	320				1.84% (0.79%, 2.51%)	U = 11808.00, z = -0.86	.431	r = .05
Not Bothered	277	86.56%	88.1%	85.57%				
A Little Bothered	29	9.06%	11.9%	7.22%				
A Lot Bothered	14	4.38%	0%	7.22%				

Note. N = 326. Percentages are calculated within the respective overall sample or gender group. Gender differences were analyzed using Mann–Whitney U tests (r: .10 = small, .24 = medium, .37 = large). Mann–Whitney z values include tie and continuity corrections; r uses tie correction only. p values were Benjamini–Hochberg-adjusted across the ten stressor comparisons. All tests were two-tailed, with $p < .05$ as the criterion for statistical significance. Boldface indicates significant gender differences.

Supplement Table 4

Correlations of Depressive Symptoms and Psychosocial Stressor Items

Variable	Overall	Men	Women	Difference
Health Concerns	.52**	.45**	.56**	.11
Appearance Concerns	.38**	.29**	.41**	.12
Lack of Sexual Desire/Enjoyment	.41**	.25**	.49**	.24(*)
Relationship Issues	.46**	.39**	.51**	.12
Family Caregiving Stress	.48**	.51**	.46**	-.05
Occupational Stress	.29**	.32**	.25**	-.07
Financial Concerns	.49**	.52**	.46**	-.06
Lack of Support	.49**	.34**	.56**	.22(*)
Traumatic Memories	.47**	.40**	.49**	.09
Recent Adverse Event	.43**	.26**	.52**	.26**

Note. N = 326. Pairwise sample sizes vary because correlations were calculated using complete observations for each variable pair. Spearman rank correlations. Difference values represent women's correlation minus men's correlation. The permutation test was used to compute group differences with 1,000 permutations. *$p < .05$; **$p < .01$. Asterisks in parentheses indicate that the result is no longer significant after applying the Benjamini–Hochberg correction for multiple comparisons.

Supplement Table 5

Mediation of the Gender Differences in Depressive Symptoms Through Key Variables

Mediator Path	Estimate	95% CI *LL*	95% CI *UL*	*SE*	*p*
Occupational status					
a	0.164	-0.092	0.419	0.130	.210
b	-0.516	-0.883	-0.150	0.187	.006
Indirect	-0.084	-0.252	0.083	0.086	.324
Education Germany					
a	0.366	0.068	0.664	0.152	.016
b	0.208	-0.175	0.590	0.195	.288
Indirect	0.076	-0.077	0.229	0.078	.331
Financial status					
a	0.461	0.199	0.723	0.134	< .001
b	-0.042	-0.181	0.097	0.071	.555
Indirect	-0.019	-0.085	0.046	0.033	.563
Age at migration					
a	-0.339	-0.581	-0.098	0.123	.006
b	0.136	-0.060	0.331	0.100	.174
Indirect	-0.046	-0.120	0.028	0.038	.224
German language proficiency					
a	-0.355	-0.597	-0.114	0.123	.004
b	-0.035	-0.319	0.250	0.145	.811
Indirect	0.012	-0.089	0.114	0.052	.812
German cultural orientation					
a	0.407	0.184	0.629	0.113	< .001
b	-0.045	-0.146	0.055	0.051	.377
Indirect	-0.018	-0.060	0.024	0.021	.392
Reason for Migration Refugee					
a	0.114	0.018	0.210	0.049	.019
b	-0.222	-0.695	0.250	0.241	.357
Indirect	-0.025	-0.083	0.033	0.030	.392
Reason for Migration Family					
a	-0.165	-0.355	0.024	0.097	.087
b	0.120	-0.111	0.350	0.118	.308
Indirect	-0.020	-0.064	0.024	0.022	.373
Years in Germany					
a	0.327	0.104	0.551	0.114	.004
b	0.066	-0.070	0.202	0.069	.342
Indirect	0.022	-0.025	0.068	0.024	.365
PHQ Psychosocial Stress					
a	-0.370	-0.641	-0.099	0.138	.008
b	0.742	0.637	0.848	0.054	< .001
Indirect	-0.274	-0.480	-0.069	0.105	.009

Note. $N = 326$; WLSMV used pairwise available observations. *a* = gender-to-mediator path; *b* = mediator-to-depressive-symptoms path. Unstandardized estimates and robust 95% CIs are shown. CI = confidence interval; *LL* = lower limit; *UL* = upper limit. Fit (scaled except SRMR): $\chi^2(22) = 47.05$, $p = .001$, CFI = .955, TLI = .888, RMSEA = .059, 90% CI [.036, .083], SRMR = .059. Overall R^2 for depressive symptoms = .58.

Supplement Table 6

Oaxaca–Blinder Decomposition of the Gender Difference in Depressive Symptoms

Panel A. Twofold decomposition

**Model**	**Observed gap [95% CI]**	**Characteristics [95% CI] (% of gap)**	**Coefficients [95% CI]**
Primary decomposition	1.51 [0.63, 2.40]	1.47 [0.69, 2.24] (97.0%)	0.05 [−0.69, 0.78]

Panel B. Contributions to the characteristics component in the primary model

**Characteristic**	**Contribution [95% CI]**
Occupational status	0.12 [−0.04, 0.28]
Education in Germany	−0.08 [−0.23, 0.08]
Financial status	0.13 [−0.08, 0.34]
Age at migration	−0.07 [−0.23, 0.10]
German language proficiency	−0.05 [−0.23, 0.12]
German cultural orientation	0.12 [−0.08, 0.31]
Reason for migration: refugee	0.06 [−0.06, 0.17]
Reason for migration: family	0.05 [−0.10, 0.19]
Years in Germany	−0.08 [−0.22, 0.06]
Psychosocial stress	1.28 [0.60, 1.95]

Note. n = 321 (126 men and 195 women). Five participants without a valid PHQ-9 score were excluded. The observed gap was calculated as the mean PHQ-9 score among women minus that among men; all estimates are expressed in PHQ-9 points. The characteristics component reflects the component associated with differences between women and men in the distributions of the included characteristics, whereas the coefficients component reflects the component associated with differences in their estimated associations with depressive symptoms. Positive contributions in Panel B indicate that group differences in a characteristic increased the observed gap; negative contributions indicate attenuation. The primary decomposition used coefficients from a pooled regression including gender. Occupational status represents working versus not working. Reason for migration was represented by family and refugee indicators, with work, education, or other reasons as the reference category. Missing predictor data were multiply imputed separately by gender (m = 50; 20 iterations). Within-imputation uncertainty was estimated using 1,000 stratified bootstrap samples, and estimates were combined using Rubin's rules. Across alternative reference structures, estimates for the characteristics component ranged from 1.37 to 1.53 points, with all corresponding 95% CIs excluding zero; all 95% CIs for the coefficients component included zero. CI = confidence interval

Oaxaca–Blinder Decomposition

To complement the mediation analysis, a twofold Oaxaca–Blinder decomposition was conducted using the same predictors as the retained mediation model. The characteristics component was 1.47 PHQ-9 points (95% CI [0.69, 2.24]), corresponding to 97.0% of the observed mean difference, whereas the coefficients component was 0.05 points (95% CI [−0.69, 0.78]). Results were substantively unchanged when group-specific coefficients, pooled coefficients excluding gender, equal group weights, or sample-size weights were used as alternative reference structures: characteristics component estimates ranged from 1.37 to 1.53 points, with all corresponding confidence intervals excluding zero, whereas all confidence intervals for the coefficients component included zero. Psychosocial stress made the largest individual contribution (1.28 points, 95% CI [0.60, 1.95]). These descriptive findings indicate that the observed difference was predominantly associated with group differences in the included characteristics rather than differences in their estimated coefficients; they do not establish causal mediation.
